# Significance of a preoperative systemic immune-inflammation index as a predictor of postoperative survival outcomes in gastric cancer

**DOI:** 10.1186/s12957-021-02286-3

**Published:** 2021-06-12

**Authors:** Hiroyuki Inoue, Toshiyuki Kosuga, Takeshi Kubota, Hirotaka Konishi, Atsushi Shiozaki, Kazuma Okamoto, Hitoshi Fujiwara, Eigo Otsuji

**Affiliations:** 1 Division of Digestive Surgery, Department of Surgery, Kyoto Prefectural University of Medicine, 465 Kajii-cho, Kamigyo-ku, Kyoto, 602-8566 Japan; 2 Department of Surgery, Saiseikai Shiga Hospital, Ritto, Shiga Japan

**Keywords:** Gastric cancer, Systemic immune-inflammation index, Gastrectomy, Prognosis

## Abstract

**Background:**

Since inflammation and the immune system contribute to the development and progression of malignancies, parameters that reflect a host’s immune-inflammatory status may be useful prognostic indicators of gastric cancer (GC). The present study examined the clinical significance of a preoperative systemic immune-inflammation index (SII) for predicting postoperative survival outcomes in GC.

**Methods:**

A total of 447 patients who underwent curative gastrectomy for GC were included in the present study. SII was calculated as platelet count × neutrophil count/lymphocyte count. The prognostic impact of preoperative SII was examined using univariate and multivariate analyses.

**Results:**

Preoperative SII ranged between 105 and 4455 (median 474), and the optimal cutoff value for predicting overall survival (OS) was 395 based on a receiver operating characteristic curve. The 5-year OS rate of the SII ≥ 395 group was 80.0%, which was significantly worse than that (92.7%) of the SII < 395 group (*p* < 0.001). The multivariate analysis identified SII ≥ 395 (hazard ratio [HR] 2.95; 95% confidence interval [CI] 1.49–6.39; *p* = 0.001), heart disease (HR 2.14, 95% CI 1.07–4.07), C-reactive protein ≥ 0.5 (HR 2.45, 95% CI 1.15–4.94), pT4 (HR 4.46, 95% CI 2.44–8.14), and pN+ (HR 4.02, 95% CI 2.10–7.93) as independent predictors of worse OS. Peritoneal recurrence was more frequent in the high SII group than in the low SII group (*p* = 0.028).

**Conclusion:**

Preoperative SII may be a useful predictor of postoperative survival outcomes in GC. The meticulous surveillance of GC relapse, particularly peritoneal dissemination, is necessary for patients with SII ≥ 395 even after curative gastrectomy.

**Supplementary Information:**

The online version contains supplementary material available at 10.1186/s12957-021-02286-3.

## Background

Gastric cancer (GC) is the sixth most common cancer and the second leading cause of cancer-related death worldwide [1]. Despite the recent advances in surgical procedures and chemotherapy, recurrence is still reported in a large number of patients with GC even after curative resection. Therefore, to improve the survival outcomes of GC, it is important to identify patients with a high risk of GC recurrence who need to be treated with adequate adjuvant treatments and meticulous surveillance even after curative gastrectomy.

Since inflammation and the immune system contribute to the development and progression of malignancies [2, 3], parameters that reflect a host’s immune-inflammatory status may be useful prognostic indicators of GC. Neutrophil (Neut), lymphocyte (Lymp), monocyte (Mono), and platelet (Plt) counts in the peripheral blood were previously shown to be convenient and helpful prognostic indicators of GC [4–6]. A systemic immune-inflammation index (SII), readily calculated by the combination of three complete blood count (CBC) parameters (Neut, Lymp, and Plt), has recently been attracting increasing attention as a powerful prognostic indicator in several malignancies, such as esophageal, colon, and pancreatic cancers [7–9]; however, the clinical utility of SII in GC remains unclear.

In the present study, the clinical significance of preoperative SII for predicting the postoperative survival outcomes of GC was compared with that of each CBC parameter (Neut, Lymp, Mono, or Plt). The relationship between preoperative SII and the type of GC recurrence was also examined. The aim of the present study was to establish a novel perioperative care system according to preoperative SII levels.

## Methods

### Patients

A total of 447 patients who underwent surgery for GC between January 2008 and June 2013 at the Kyoto Prefectural University of Medicine were included in the present study. The inclusion criteria were as follows: (1) patients pathologically diagnosed with gastric adenocarcinoma; (2) patients with pathological stages (pStage) I, II, and III; and (3) patients undergoing the curative resection of GC (R0). Patients who received neoadjuvant chemotherapy; those who underwent emergency surgery; those who underwent non-curative resection (R1/R2); those with hematological, chronic inflammatory, or autoimmune diseases that may affect preoperative SII values; and those with active infection or inflammatory diseases within 1 month before the blood examination were excluded. Patients with missing information on preoperative CBC and those with simultaneous malignancies other than GC were also excluded. Gastrectomy with lymph node dissection was performed based on the Japanese Gastric Cancer Treatment Guidelines (JGCTG) [10]. Tumor staging was performed according to the 8th edition of the Tumor, Node, Metastasis staging classification by the Union for International Cancer Control [11]. In the present study, grade 2 or higher postoperative complications according to the Clavien-Dindo Classification [12, 13] occurred in 60 patients (13.4%), and only one patient (0.22%) died of postoperative complications.

All patients provided written informed consent before surgery. The present study was approved by the Ethics Committee of the Kyoto Prefectural University of Medicine (approval no. ERB-C-1327).

### Data collection of preoperative CBC and SII

Preoperative CBC (Neut, Lymp, Mono, and Plt) was collected from patients within 7 days before surgery, and SII was calculated as Plt (cell/mm^3^ × 10^3^) × Neut (cell/mm^3^)/Lymp (cell/mm^3^).

### Clinicopathological findings

The following clinicopathological data were reviewed from the medical record database of our institution: age, sex, body mass index (BMI), physical status (PS), comorbidities (hypertension, diabetes mellitus, heart disease, and chronic renal failure), tumor location, preoperative serum carcinoembryonic antigen (CEA), cancer antigen (CA) 19-9, albumin, C-reactive protein (CRP), preoperative CBC (Neut, Lymp, Mono, and Plt), pathological T stage (pT), pathological N stage (pN), lymphatic invasion, venous invasion, and tumor differentiation.

### Adjuvant treatment and postoperative follow-up

After curative gastrectomy for GC, patients with pStage I generally received postoperative examinations alone without any adjuvant treatments. Patients with pStage II and III were basically treated with adjuvant chemotherapy based on JGCTG. The main chemotherapeutic regimens administered were S-1 alone [14], S-1 plus oxaliplatin (SOX) [15], or capecitabine plus oxaliplatin (CapeOX) [16], in line with JGCTG [10]. All patients were followed up at regular intervals with serum CEA and CA19-9; computed tomography (CT) of the chest, abdomen, and pelvis; and upper gastrointestinal endoscopy following the JGCTG. Most patients were postoperatively followed up for 5 years or until their death. GC recurrence was confirmed by imaging, such as CT and upper gastrointestinal endoscopy. If possible, recurrence was histologically confirmed via surgical biopsy, needle biopsy, or appropriate fluid cytology. Peritoneal recurrence was diagnosed by imaging alone, and diagnostic laparotomy was rarely performed.

### Statistical analysis

Differences between the two groups for categorical and continuous variables were analyzed by the chi-squared test and Mann-Whitney U test, respectively. The optimal cutoff value for each immune-nutritional parameter (Neut, Lymp, Mono, Plt, or SII) was selected according to the receiver operating characteristic (ROC) curve for overall survival (OS) with the maximal Youden index based on the sum of sensitivity and specificity [17, 18]. The cutoff values for serum albumin and CRP were set at 3.5 g/dl and 0.5 mg/dl, respectively, with reference to the modified Glasgow Prognostic Score, which is regarded as a useful prognostic indicator in various malignancies [19, 20]. OS and recurrence-free survival (RFS) were generated using the Kaplan-Meier method, and the differences between the two groups were assessed with the log-rank test. Parameters showing significance in univariate analyses were further assessed using multivariate Cox’s models. We used two types of multivariate Cox’s models. In one model (model #1), Neut, Lymp, or Plt was separately incorporated as explanatory variables. In the other model (model #2), SII was incorporated as an explanatory variable instead of Neut, Lymp, and Plt. Hazard ratios (HR) and 95% confidence intervals (CI) were subsequently calculated. A *p* value < 0.05 was considered to be significant. Statistical analyses were performed with the software package JMP software version 10 (JMP, Cary, NC, USA).

## Results

### Clinical characteristics

The clinicopathological characteristics of 447 patients are shown in Table 1. The median age was 67 years (range 29–89), with 289 male (64.7%) and 158 female patients (35.3%). The median (range) values were 3690 (1250–15,850) for Neut, 1780 (570–4770) for Lymp, 330 (50–1400) for Mono, 22.5 × 10^4^ (8.7 × 10^4^–57.0 × 10^4^) for Plt, and 474 (105–4455) for SII.

**Table 1:** Clinicopathological characteristics of the patients

Variables		All patients (*n* = 447)	SII > 395 (*n* = 280)	SII < 395 (*n* = 167)	*p* value
Age, years	Median	67	67	67	0.215
	Range	29–89	29–89	39–89	
Sex	Male	289	176 (62.9%)	113 (67.7%)	0.302
	Female	158	104 (37.1%)	54 (32.3%)	
BMI, kg/m^2^	Median	22.2	21.9	23.1	0.021*
	Range	14.4–34.2	14.4–34.2	15.6–30.6	
PS	1	233	146 (52.1%)	87 (52.1%)	0.992
	> 2	214	134 (47.9%)	80 (47.9%)	
Hypertension	Presence	114	77 (27.5%)	37 (22.2%)	0.210
	Absence	333	203 (72.5%)	130 (77.8%)	
Diabetes mellitus	Presence	64	35 (12.5%)	29 (17.4%)	0.155
	Absence	383	245 (87.5%)	138 (82.6%)	
Heart disease	Presence	50	30 (10.7%)	20 (12.0%)	0.682
	Absence	397	250 (89.3%)	147 (88.0%)	
Chronic renal failure	Presence	10	6 (2.1%)	4 (2.4%)	0.862
	Absence	437	274 (97.9%)	163 (97.6%)	
Tumor location	Upper	145	96 (34.3%)	49 (29.3%)	0.278
	Middle/lower	302	184 (65.7%)	118 (70.7%)	
CEA, ng/ml	< 5	390	240 (85.7%)	150 (89.8%)	0.202
	≥ 5	57	40 (14.3%)	17 (10.2%)	
CA19-9, U/ml	< 37	422	261 (93.2%)	161 (96.4%)	0.143
	≥ 37	25	19 (6.8%)	6 (3.6%)	
Albumin, g/dl	< 3.5	19	17 (6.1%)	2 (1.2%)	0.014*
	≥ 3.5	428	263 (93.9%)	165 (98.8%)	
CRP, mg/dl	< 0.5	397	245 (87.5%)	152 (91.0%)	0.254
	≥ 0.5	50	35 (12.5%)	15 (9.0%)	
Neutrophil count, cell/mm^3^	Median	3690	4145	3060	< 0.001*
	Range	1250–15,850	1250–15,850	1420–7630	
Lymphocyte count, cell/mm^3^	Median	1780	1630	2050	< 0.001*
	Range	570–4770	570–4770	1050–4640	
Monocyte count, cell/mm^3^	Median	330	340	310	0.008*
	Range	50–1400	50–1400	100–750	
Platelet count, cell/mm^3^ × 10^4^	Median	225	245	190	< 0.001*
	Range	87–570	570–117	87–314	
pT	pT1–3	399	248 (88.6%)	151 (90.4%)	0.539
	pT4	48	32 (11.4%)	16 (9.6%)	
pN	pN0	323	189 (67.5%)	134 (80.2%)	0.003*
	pN+	124	91 (32.5%)	33 (19.8%)	
Lymphatic invasion	Presence	169	120 (42.9%)	49 (29.3%)	0.004*
	Absence	278	160 (57.1%)	118 (70.6%)	
Venous invasion	Presence	138	95 (33.9%)	43 (25.7%)	0.068
	Absence	309	185 (66.1%)	124 (74.3%)	
Tumor differentiation	Differentiated	231	133 (47.5%)	98 (58.7%)	0.022*
	Undifferentiated	216	147 (52.5%)	69 (41.3%)	

*BMI* body mass index, *PS* physical status, *CEA* carcinoembryonic antigen, *CA19-9* cancer antigen 19-9, *CRP* C-reactive protein, *SII* systemic immune inflammation index

^*^p < 0.05 (significantly different between the high and low SII groups)

### ROC curve analysis

**Fig. 1:**
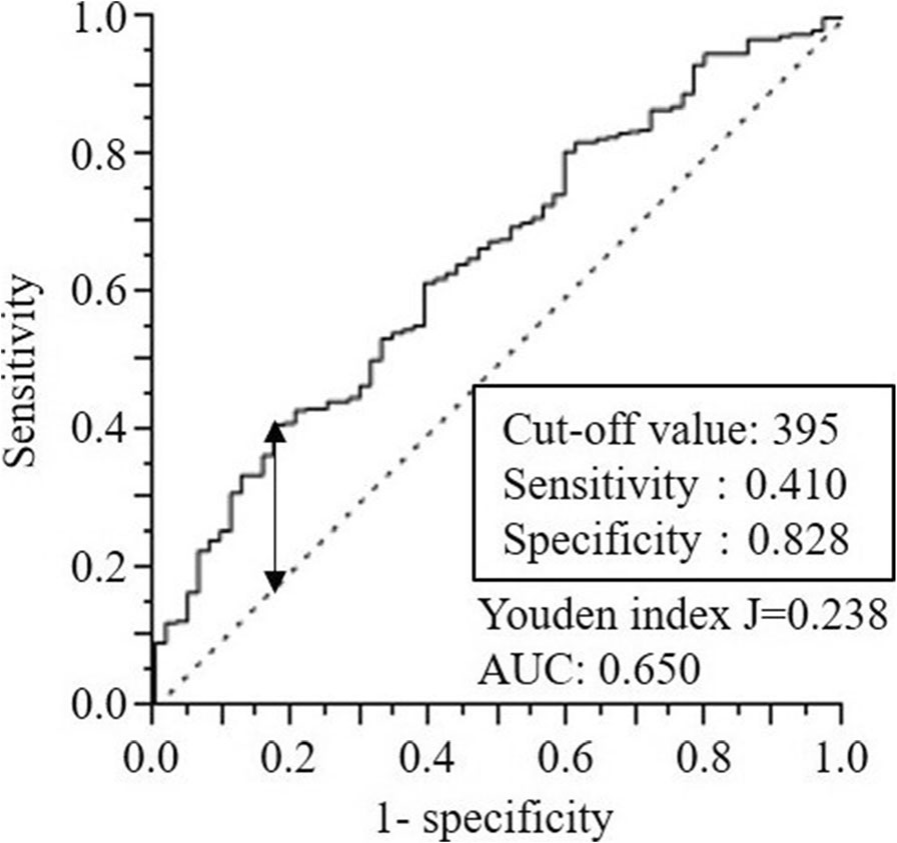
ROC curve analysis of preoperative SII for predicting OS in patients with GC

The area under the curve (AUC) value showing the predictive power of SII for 5-year OS was 0.650, and the Youden index was 0.238. As shown in Fig. 1, the optimal cutoff value for preoperative SII was 395 (sensitivity 41.0%, specificity 82.8%). All patients were then divided into the high SII (SII ≥ 395) and low SII (SII < 395) groups. The optimal cutoff values of other preoperative CBC parameters for predicting 5-year OS are shown in Additional file 1: Figure S1 (3690 for Neut (AUC 0.612), 1860 for Lymp (AUC 0.587), 320 for Mono (AUC 0.523), and 27.2 × 10^4^ for Plt (AUC 0.581)).

### Relationship between preoperative SII and clinicopathological factors

The differences in clinicopathological characteristics between the high and low SII groups are shown in Table 1. Preoperative Neut, Lymp, Mono, and Plt were significantly higher (*p* < 0.001, < 0.001, = 0.008, and < 0.001, respectively), and BMI was significantly lower (*p* = 0.021) in the high SII group than in the low SII group. In addition, hypoalbuminemia (albumin < 3.5), pN+, positive lymphatic invasion, and undifferentiated adenocarcinoma were frequent among patients in the high SII group (*p* = 0.014, 0.003, 0.004, and 0.022, respectively). No significant differences were observed in age, sex, PS, comorbidities, tumor location, preoperative CEA, CA 19-9, and CRP, pT, or venous invasion between the two groups.

### Prognostic significance of preoperative SII

**Fig. 2:**
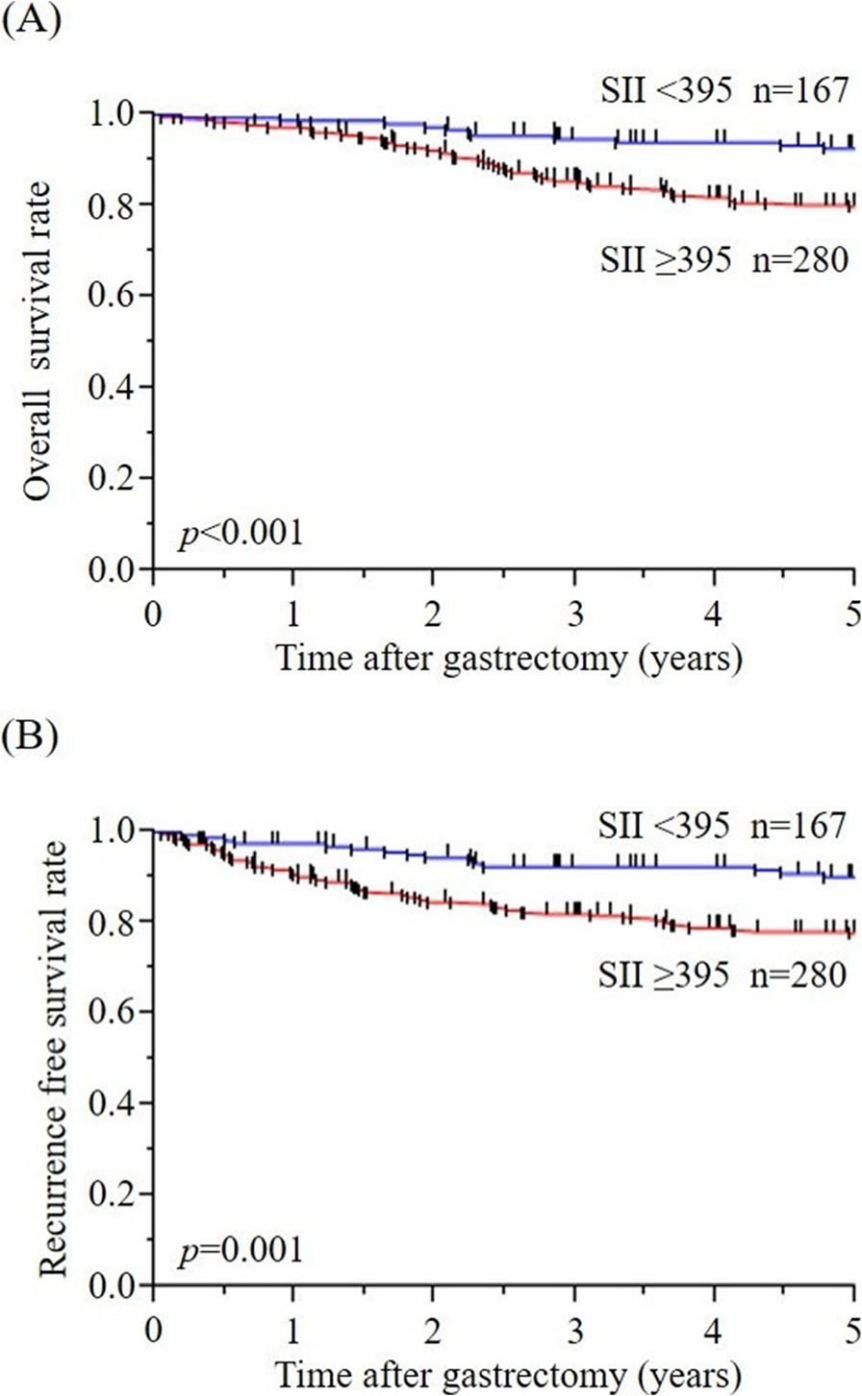
Survival curves of patients stratified by preoperative SII. **A** OS. **B** RFS

Survival curves for OS stratified by preoperative SII and each CBC parameter (Neut, Lymp, Mono, and Plt) are shown in Fig. 2A and Additional file 2: Figure S2, respectively. The 5-year OS rate of the high SII group was 80.0%, which was significantly worse than that (92.7%) of the low SII group (*p* < 0.001). Univariate and multivariate survival analyses of OS are shown in Table 2. In the univariate analysis, PS ≥ 2, hypertension, heart disease, upper GC, CEA ≥ 5, CA19-9 ≥ 37, albumin< 3.5, CRP ≥ 0.5, pT4, pN+, positive lymphatic invasion, positive venous invasion, Neut ≥ 3690, Lymp < 1860, Plt ≥ 27.2 × 10^4^, and SII ≥ 395 correlated with poor OS. In multivariate Cox’s model #1, Neut ≥ 3690 (HR 1.97; 95% CI 1.08–3.68; *p* = 0.027), heart disease, CRP ≥ 0.5, pT4, and pN+ were identified as independent risk factors for poor OS. In multivariate Cox’s model #2, SII ≥ 395 (HR 2.95; 95% CI 1.49–6.39; *p* = 0.001), heart disease, CRP ≥ 0.5, pT4, and pN+ were identified as independent risk factors for poor OS.

**Table 2:** Univariate and multivariate survival analyses for OS

Variables		All patients (*n* = 447)	Univariate		Multivariate (model #1)		Multivariate (model #2)	
			5-year OS (%)	*p* value	HR (95% CI)	*p* value	HR (95% CI)	*p* value
Age, years	< 65	184	86.9	0.262				
	> 65	263	82.9					
Sex	Male	289	84.9	0.846				
	Female	158	84.0					
BMI, kg/m^2^	< 25	353	84.3	0.701				
	> 25	94	85.7					
PS	1	233	89.4	0.004*	1 1.16 (0.66–2.06)	0.601	1 1.06 (0.61–1.86)	0.837
	> 2	214	78.8					
Hypertension	Presence	114	76.2	< 0.001*	1.50 (0.85–2.58) 1	0.158	1.50 (0.87–2.54) 1	0.147
	Absence	333	87.4					
Diabetes mellitus	Presence	64	79.5	0.177				
	Absence	383	85.4					
Heart disease	Presence	50	70.0	< 0.001*	2.52 (1.22–4.99) 1	0.014*	2.14 (1.07–4.07) 1	0.033*
	Absence	397	86.4					
Chronic renal failure	Presence	10	77.1	0.384				
	Absence	437	84.8					
Tumor location	Upper	145	75.5	< 0.001*	1.60 (0.93–2.71) 1	0.086	1.53 (0.90–2.58) 1	0.117
	Middle/lower	302	88.7					
CEA, ng/ml	< 5	390	86.1	0.008*	1 1.51 (0.74–2.85)	0.243	1 1.68 (0.83–3.15)	0.143
	≥ 5	57	72.4					
CA19-9, U/ml	< 37	422	85.7	0.003*	1 1.64 (0.77–3.90)	0.206	1 1.46 (0.70–3.42)	0.328
	≥ 37	25	65.3					
Albumin, g/dl	< 3.5	19	44.2	< 0.001*	1.11 (0.45–2.92) 1	0.827	1.07 (0.40–2.31) 1	0.872
	≥ 3.5	428	86.4					
CRP, mg/dl	< 0.5	397	87.0	< 0.001*	1 2.51 (1.16–5.16)	0.021*	1 2.45 (1.15–4.94)	0.021*
	≥ 0.5	50	65.5					
Neutrophil count, cell/mm^3^	< 3690	223	88.9	0.020*	1 1.97 (1.08–3.68)	0.027*		NA
	≥ 3690	224	80.3					
Lymphocyte count, cell/mm^3^	≥ 1860	200	89.3	0.011*	1 1.41 (0.80–2.56)	0.242		NA
	< 1860	247	80.6					
Monocyte count, cell/mm^3^	< 320	194	87.4	0.198				
	≥ 320	253	82.4					
Platelet count, cell/mm^3^ × 10^4^	< 27.2	338	87.6	0.003*	1 1.25 (0.65–2.34)	0.494		NA
	≥ 27.2	109	75.2					
SII	< 395	167	92.7	< 0.001*		NA	1 2.95 (1.49–6.39)	0.001*
	≥ 395	280	80.0					
pT	pT1-3	399	93.8	< 0.001*	1 3.99 (2.17–7.35)	< 0.001*	1 4.46 (2.44–8.14)	< 0.001*
	pT4	48	57.8					
pN	pN0	323	93.7	< 0.001*	1 3.93 (2.01–7.95)	< 0.001*	1 4.02 (2.10–7.93)	< 0.001*
	pN+	124	60.8					
Lymphatic invasion	Presence	169	71.3	< 0.001*	1.35 (0.69–2.68) 1	0.386	1.18 (0.61–2.32) 1	0.626
	Absence	278	92.7					
Venous invasion	Presence	138	71.2	< 0.001*	1.13 (0.65–1.97) 1	0.675	1.22 (0.70–2.14) 1	0.475
	Absence	309	90.3					
Tumor differentiation	Differentiated	231	87.3	0.148				
	Undifferentiated	216	81.8					

*OS* overall survival, *BMI* body mass index*, PS* physical status, *CEA* carcinoembryonic antigen, *CA19-9* cancer antigen 19-9, *CRP* C-reactive protein, *SII* systemic immune inflammation index, *HR* hazard ratio, *CI* confidence interval, *NA* not applicable

^*^p < 0.05 (significantly different between two groups)

Similar to the results of analyses of OS, the 5-year RFS rate of the high SII group was 77.7%, which was significantly worse than that (90.2%) of the low SII group (*p* = 0.001) (Fig. 2B). The multivariate Cox’s model identified SII ≥ 395 as an independent risk predictor of 5-year RFS (HR 2.36; 95% CI 1.31–4.48; *p* = 0.004) (Additional file 3: Table S1).

### Relationship between preoperative SII and the type of GC recurrence

**Fig. 3:**
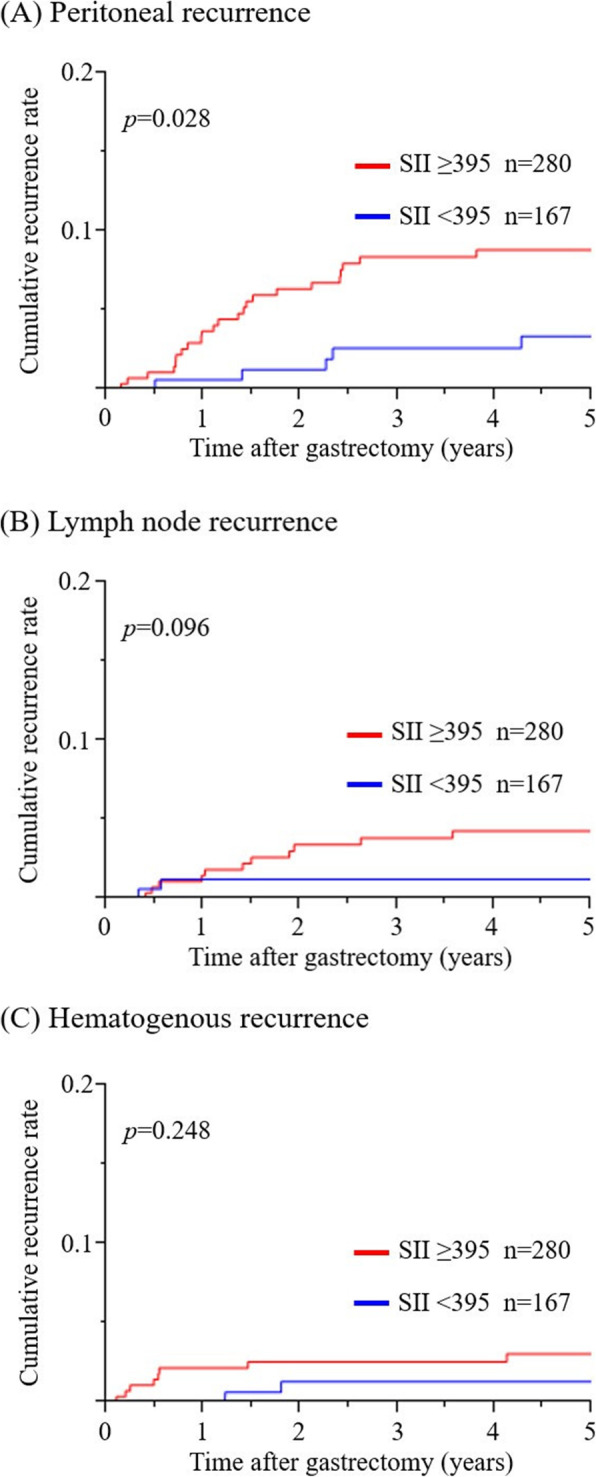
Cumulative recurrence rate for each recurrence pattern stratified by preoperative SII. **A** Peritoneal recurrence. **B** Lymph node recurrence. **C** Hematogenous recurrence

GC recurrence was detected in 51 out of 447 patients. Cumulative recurrence rates stratified by preoperative SII were examined according to the type of GC recurrence (peritoneal, hematogenous, and lymph node recurrence) (Fig. 3). Peritoneal recurrence was more frequent in the high SII group than in the low SII group (*p* = 0.028), whereas no significant difference was observed in the cumulative recurrence rate for hematogenous or lymph node recurrence between the high and low SII groups (*p* = 0.248 and 0.096, respectively).

## Discussion

In the present study, we examined the clinical significance of preoperative SII to predict postoperative survival outcomes in GC. The optimal cutoff value of preoperative SII for predicting OS was set at 395 according to the ROC curve analysis. The results obtained demonstrated that SII, a combination of three CBC parameters (Neut, Lymp, and Plt), was an independent risk factor for poor OS and RFS as well as pT and pN. Of special note was that high SII correlated with peritoneal recurrence. These results suggested that preoperative SII may contribute to perioperative precise care and adjuvant treatments for patients with GC undergoing curative gastrectomy.

In the present study, in addition to Neut, Lymp, Mono, and Plt, preoperative serum albumin and BMI correlated with preoperative SII. A relationship was previously suggested between obesity and chronic inflammation [21]; therefore, patients with high BMI may have a stronger inflammatory response. However, the present study showed that BMI was significantly lower in the high SII group. In cancer patients, inflammation is induced by inflammatory cytokines as cancer progresses, and, thus, patients with a high inflammatory response may lose weight [22]. In the present study, high SII correlated with low albumin levels, suggesting that cachexia had an influence on the results obtained. Hirahara et al. also reported that BMI was significantly lower in the high SII group [23].

Neutrophilia, lymphopenia, and thrombocytosis each affect a host’s immune-inflammatory status and tumor progression and have been implicated in poor cancer survival [24–29]. In addition, several indexes calculated by combining these factors, such as neutrophil-to-lymphocyte ratio, platelet-to-lymphocyte ratio, and monocyte-to-lymphocyte ratio, have been used to predict survival outcomes of GC [30–32]. Accordingly, high SII, resulting from neutrophilia, lymphopenia, and thrombocytosis, may also be a useful prognostic indicator. In the present study, univariate survival analyses showed that Neut, Lymp, and Plt were significant prognostic factors in patients with GC; however, the multivariate survival analysis (model #1) only identified Neut ≥ 3690 (HR 1.97; 95% CI 1.08–3.68; *p* = 0.027) as an independent risk factor of poor OS. The multivariate survival analysis (model #2) also identified SII ≥ 395 (HR 2.95; 95% CI 1.49–6.39; *p* = 0.001) as an independent risk factor of poor OS, with a higher HR and lower *p* value than those of Neut in model #1. The AUC value showing the predictive power of SII for OS (AUC 0.650) was higher than that (AUC 0.612) of Neut.

The negative impact of postoperative complications on survival outcomes has recently been clarified [33, 34]. Accordingly, worse OS may be attributed to a higher incidence of postoperative complications. However, in the present study, the incidence of postoperative complications of Clavien-Dindo grade II or higher in the high SII group was 13.9% (39/280), which was not significantly different from that (21/167, 12.6%) in the low SII group (*p* = 0.684, data not shown). Correlations between preoperative inflammatory indices and the occurrence of postoperative complications remain controversial [35, 36], and, thus, further studies are needed to confirm these relationships.

Previous studies demonstrated the negative impact of high preoperative SII on the survival outcomes of GC [23, 37, 38]; however, the cutoff values of SII differed between these studies. Wang et al. [37] reported that the optimal cutoff value of SII for predicting OS was 660 (AUC 0.612) according to the ROC curve analysis, and SII ≥ 660 was an independent predictor of OS in GC patients. Hirahara et al. [23] showed that the optimal cutoff value of SII for predicting OS was 661.9 (AUC 0.584) according to the ROC curve analysis, and SII ≥ 661.9 was an independent prognostic indicator in GC patients, particularly in the elderly population. In the present study, although an ROC curve analysis was also used, the optimal cutoff value of SII for predicting OS was 395, which was markedly lower than 600. Differences in the pStage of GC patients may affect SII cutoff values. Although Wang et al. only targeted pStage III GC patients who had undergone R0, R1, or R2, the present study examined pStage I, II, and III patients undergoing R0 [37]. Similar to the present results, Shi et al. only targeted patients with pStage I–III GC who had undergone R0 and demonstrated that the optimal SII cutoff value was 320 (AUC 0.64) and that SII > 320 was an independent prognostic indicator in these patients [38]. Therefore, further studies are needed to validate the optimal cutoff value of SII.

The present study demonstrated that preoperative SII was a risk factor for poor RFS independent of pT and pN and that SII ≥ 395 correlated with GC recurrence, particularly peritoneal recurrence. To the best of our knowledge, this is the first study to demonstrate the impact of high SII on the specific recurrence pattern of GC. A previous study showed that neutrophils promoted the migration and invasion of GC cells by activating the ERK pathway and inducing epithelial-mesenchymal transition [27]. Other studies indicated that lymphopenia and thrombocytosis were predictive factors for peritoneal dissemination [39, 40]. In addition, Nakamura et al. [41] reported that the preoperative neutrophil-to-lymphocyte ratio was a significant independent predictor of the presence of peritoneal metastasis during staging laparoscopy in patients with advanced GC. Since high SII results from neutrophilia, lymphopenia, and thrombocytosis, it may also be a useful predictor of peritoneal recurrence. In the present study, from a pathological viewpoint, pN+ and undifferentiated adenocarcinoma were often observed in the high SII group. Peritoneal recurrence was also frequently detected in patients with pN+ (pN+, 25/124; pN−, 3/320; *p* < 0.001) and undifferentiated adenocarcinoma (differentiated type, 10/231; undifferentiated type, 18/216; *p* = 0.081); therefore, the incidence of peritoneal recurrence may be high in patients with high SII.

According to JGCTG, the current indication of postoperative adjuvant chemotherapy is assessed only by pStage (pT and pN). However, the present results suggest that patients with high preoperative SII may also be considered for adjuvant chemotherapy. In Western countries, perioperative chemotherapy (preoperative plus postoperative FLOT) is the standard treatment [42, 43]. Although postoperative adjuvant chemotherapy alone is the standard treatment in Eastern Asia, its effects are inadequate in many cases; therefore, patients with poor prognostic factors, such as high SII, may be good candidates for neoadjuvant chemotherapy. Perioperative interventions, such as immunonutrition, may be another treatment option for improving the survival outcomes of patients with high SII. However, there is currently no evidence to show that perioperative immunonutrition actually improves the long-term outcomes of GC patients. It also currently remains unclear whether preoperative immunonutrition reduces morbidity rates after gastrectomy for GC [44–46]. Xin et al. [47] has recently shown by a systematic review and a meta-analysis that enteral nutrition feeding tube support is an essential intervention to elevate patients’ immunity, depress levels of inflammation, and reduce the risk of complications after gastrectomy for GC. Therefore, further research and the development of novel treatment methods that fundamentally suppress cancer-induced inflammation and boost host immunity are desired.

The present study had several limitations. It was a retrospective study with a small sample size from one institution, which may have limited its statistical power and generated statistical biases. Furthermore, since the cutoff values of Neut, Lymp, Mono, Plt, and SII were only calculated by a mathematical method, further validations are needed to confirm whether these values are clinically meaningful and applicable to other cohorts. In addition, the present study only examined CBC parameters; however, serum immune-nutritional markers may also be useful predictors of survival outcomes in GC. Dynamic changes in the host’s immune-inflammatory status during the perioperative period may provide more important information about survival outcomes, but the present study failed to evaluate dynamic changes in the SII values [30]. Relationships between tumor microenvironment and the mechanisms of immune dysfunction should be uncovered to develop promising immunotherapy and improve survival outcomes of GC patients [48–50]; however, no molecular biological examinations have been done in this study. Nevertheless, the present study clearly demonstrated the novel potential of preoperative SII for predicting postoperative survival outcomes and recurrence patterns in GC over that of each CBC parameter (Neut, Lymp, Mono, and Plt). The results of the present study and the optimal cutoff value of SII need to be validated in further studies with large sample sizes in order to establish a novel perioperative care system according to preoperative SII levels.

## Conclusion

Preoperative SII may be a useful predictor of postoperative survival outcomes in GC. Appropriate postoperative adjuvant treatments and the meticulous surveillance of GC relapse, particularly peritoneal dissemination, are necessary for patients with SII ≥ 395 even after curative gastrectomy.

## Supplementary Information

**Additional file 1: Figure S1.** ROC curve analysis of preoperative each CBC parameter for predicting OS in patients with GC. (A) Neutrophil, (B) Lymphocyte, (C) Monocyte, (D) Platelet.

**Additional file 2: Figure S2.** OS curves of the patients stratified CBC parameter. (A) Neutrophil, (B) Lymphocyte, (A) Monocyte, (A) Platelet.

**Additional file 3: Table S1.** Univariate and multivariate survival analyses for RFS.

## Data Availability

The datasets used and/or analyzed during the current study are available from the corresponding author on reasonable request.

## Abbreviations

AUC: Area under the curve
BMI: Body mass index
CA19-9: Cancer antigen 19-9
CBC: Complete blood count
CEA: Carcinoembryonic antigen
CI: Confidence interval
CT: Computed tomography
GC: Gastric cancer
HR: Hazard ratio
JGCTG: Japanese Gastric Cancer Treatment Guidelines
Lymp: Lymphocyte
Mono: Monocyte
Neut: Neutrophil
OS: Overall survival
Plt: Platelet
PS: Physical status
RFS: Recurrence-free survival
ROC: Receiver operating characteristic
SII: Systematic immune-inflammation index
