# Supplementary figures and images for: Significance of a preoperative systemic immune-inflammation index as a predictor of postoperative survival outcomes in gastric cancer
Source: World J Surg Oncol. 2021 Jun 12;19:173. doi: 10.1186/s12957-021-02286-3 (PMC8199826; doi:10.1186/s12957-021-02286-3)

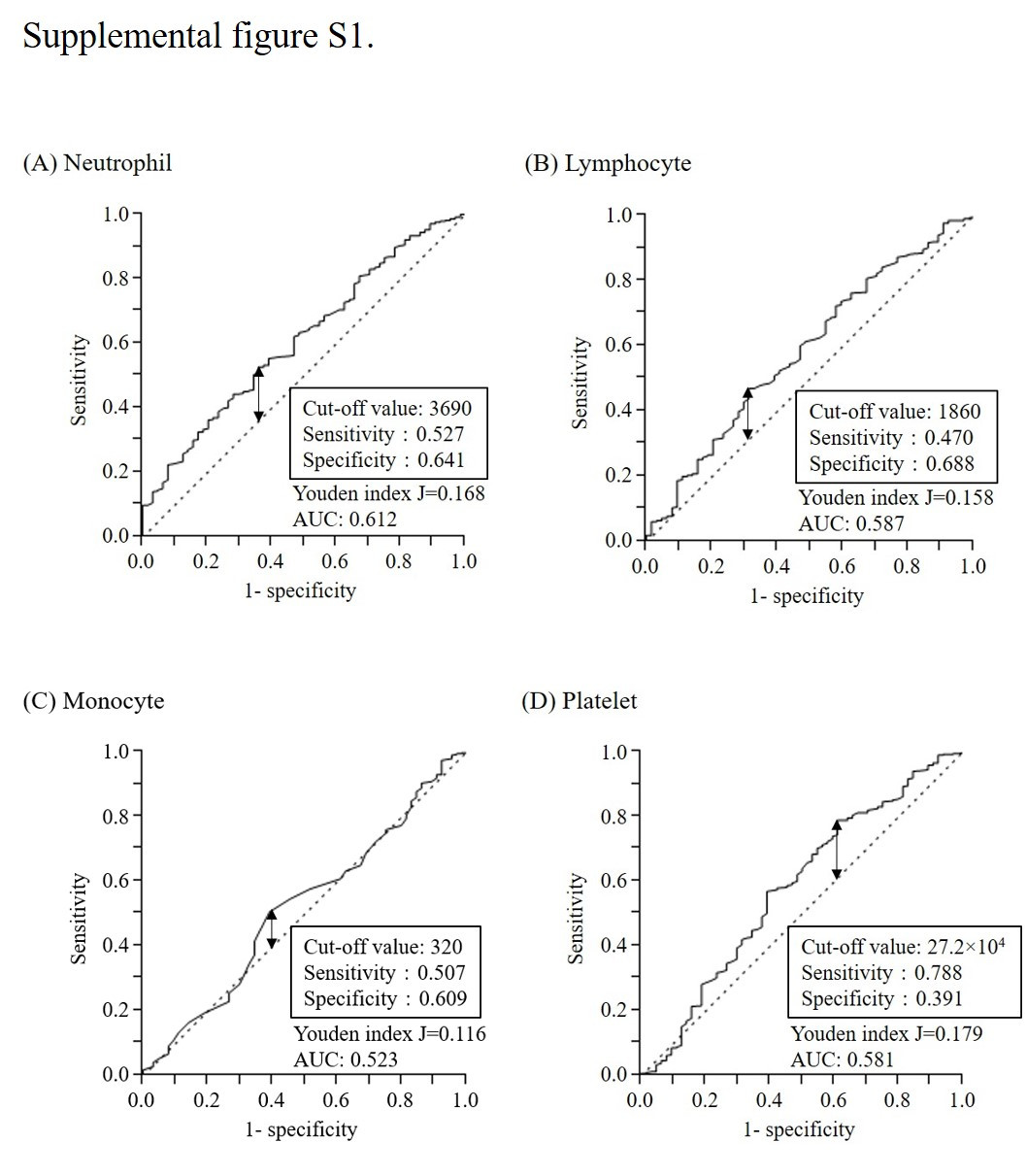

Supplement: Supplementary file 1 — Additional file 1: Figure S1. ROC curve analysis of preoperative each CBC parameter for predicting OS in patients with GC. (A) Neutrophil, (B) Lymphocyte, (C) Monocyte, (D) Platelet. [file 12957_2021_2286_MOESM1_ESM.jpg]

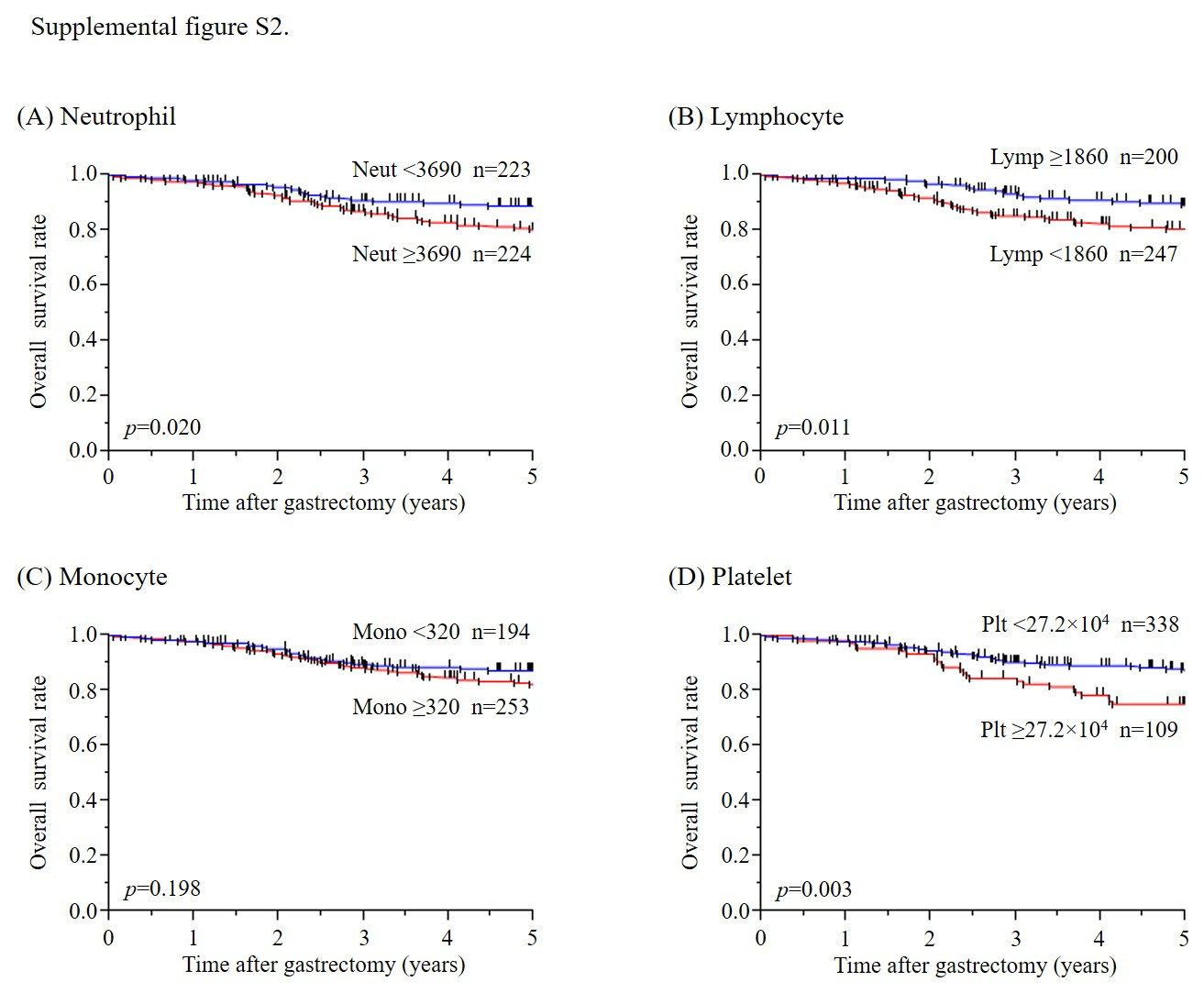

Supplement: Supplementary file 2 — Additional file 2: Figure S2. OS curves of the patients stratified CBC parameter. (A) Neutrophil, (B) Lymphocyte, (A) Monocyte, (A) Platelet. [file 12957_2021_2286_MOESM2_ESM.jpg]
