# Supplementary material for: Significance of a preoperative systemic immune-inflammation index as a predictor of postoperative survival outcomes in gastric cancer
Source: World J Surg Oncol. 2021 Jun 12;19:173. doi: 10.1186/s12957-021-02286-3 (PMC8199826; doi:10.1186/s12957-021-02286-3)
Supplement: Supplementary file 3 — Additional file 3: Table S1. Univariate and multivariate survival analyses for RFS. [file 12957_2021_2286_MOESM3_ESM.docx]

**Supplemental table S1.** Univariate and multivariate survival analyses for RFS

| Variables |  | All patients (n=447) | Univariate | | Multivariate (model #1) | | Multivariate (model #2) | |
| --- | --- | --- | --- | --- | --- | --- | --- | --- |
|  |  |  | 5-year RFS (%) | *p* value | HR (95%CI) | *p* value | HR (95%CI) | *p* value |
| Age, years | <65  >65 | 184  263 | 85.3  80.1 | 0.155 |  |  |  |  |
| Sex | Male  Female | 289  158 | 82.3  82.3 | 0.977 |  |  |  |  |
| BMI, kg/m^2^ | <25  >25 | 353  94 | 81.6  84.9 | 0.455 |  |  |  |  |
| PS | 1  >2 | 233  214 | 85.9  78.1 | 0.032* | 1  1.01 (0.61-1.69) | 0.958 | 1  1.06 (0.61-1.86) | 0.781 |
| Hypertension | Presence  Absence | 114  333 | 76.8  84.2 | 0.068 |  |  |  |  |
| Diabetes mellitus | Presence  Absence | 64  383 | 76.4  83.3 | 0.142 |  |  |  |  |
| Heart disease | Presence  Absence | 50  397 | 71.9  83.6 | 0.032* | 1.60 (0.78-3.10)  1 | 0.194 | 1.39 (0.70-2.58)  1 | 0.332 |
| Chronic renal failure | Presence  Absence | 10  437 | 77.1  82.4 | 0.637 |  |  |  |  |
| Tumor location | Upper  Middle/Lower | 145  302 | 71.0  87.6 | <0.001* | 1.95 (1.21-3.15)  1 | 0.006* | 1.89 (1.18-3.03)  1 | 0.008* |
| CEA, ng/ml | <5  ≥5 | 390  57 | 83.9  69.9 | 0.007* | 1  1.65 (0.86-2.98) | 0.131 | 1  1.76 (0.91-3.19) | 0.088 |
| CA19-9, U/ml | <37  ≥37 | 422  25 | 84.4  46.0 | <0.001* | 1  1.10 (0.54-2.11) | 0.788 | 1  1.28 (0.64-2.44) | 0.471 |
| Albumin, g/dl | <3.5  ≥3.5 | 19  428 | 44.4  84.0 | <0.001* | 1.58 (0.65-4.07)  1 | 0.315 | 1.50 (0.65-3.74)  1 | 0.354 |
| CRP, mg/dl | <0.5  ≥0.5 | 397  50 | 85.0  62.2 | <0.001* | 1  3.23 (1.54-6.45) | 0.002* | 1  3.15 (1.54-6.13) | 0.002* |
| Neutrophil count, cell/mm^3^ | <3690  ≥3690 | 223  224 | 87.1  77.6 | 0.010* | 1  1.93 (1.12-3.38) | 0.017* |  | NA |
| Lymphocyte count, cell/mm^3^ | ≥1860  <1860 | 200  247 | 86.9  78.5 | 0.018* | 1  1.25 (0.75-2.12) | 0.391 |  | NA |
| Monocyte count, cell/mm^3^ | <320  ≥320 | 194  253 | 86.5  79.1 | 0.056 |  |  |  |  |
| Platelet count, cell/mm^3^ ×10^4^ | <27.2  ≥27.2 | 338  109 | 85.6  72.1 | 0.002* | 1  1.12 (0.63-1.95) | 0.695 |  | NA |
| SII | <395  ≥395 | 167  280 | 90.2  77.7 | 0.001* |  | NA | 1  2.36 (1.31-4.48) | 0.004* |
| pT | pT1-3  pT4 | 399  48 | 92.6  52.4 | <0.001* | 1  3.37 (1.91-5.92) | <0.001* | 1  3.78 (2.15-6.63) | <0.001* |
| pN | pN0  pN+ | 323  124 | 93.1  54.2 | <0.001* | 1  4.61 (2.47-8.87) | <0.001* | 1  4.47 (2.43-8.45) | <0.001* |
| Lymphatic invasion | Presence  Absence | 169  278 | 66.9  91.6 | <0.001* | 1.56 (0.83-2.98)  1 | 0.164 | 1.43 (0.78-2.68)  1 | 0.254 |
| Venous invasion | Presence  Absence | 138  309 | 67.2  88.8 | <0.001* | 1.06 (0.63-1.82)  1 | 0.817 | 1.10 (0.65-1.88)  1 | 0.722 |
| Tumor differentiation | Differentiated  Undifferentiated | 231  216 | 84.7  79.8 | 0.207 |  |  |  |  |

RFS recurrence free survival, *BMI* body mass index*, PS* physical status, *CEA* carcinoembryonic antigen, *CA19-9* cancer antigen (CA) 19-9, *CRP* C-reactive protein, *SII* systemic immune inflammation index, *HR* hazard ratio, *CI* confidence interval, *NA* not applicable
